# High-yield production of aromatic peroxygenase by the agaric fungus *Marasmius rotula*

**DOI:** 10.1186/2191-0855-1-31

**Published:** 2011-10-11

**Authors:** Glenn Gröbe, René Ullrich, Marek J Pecyna, Danuta Kapturska, Stephanie Friedrich, Martin Hofrichter, Katrin Scheibner

**Affiliations:** 1 Department of Biology, Chemistry and Process Technology, Lausitz University of Applied Sciences, Großenhainer Straße 57, 01968 Senftenberg, Germany; 2 Department of Environmental Biotechnology, Department of Bio- and Environmental Sciences, International Graduate School of Zittau, Markt 23, 02763 Zittau, Germany; 3 Department of Soil Ecology, Helmholtz Centre for Environmental Research - UFZ Halle, Theodor-Lieser-Straße 4, 06120 Halle/Saale, Germany; 4 JenaBios GmbH, Orlaweg 2, 07743 Jena, Germany

**Keywords:** Peroxidase, Basidiomycota, Cytochrome P450, Bioreactor

## Abstract

An extracellular peroxygenase from *Marasmius rotula *was produced in liquid culture, chromatographically purified and partially characterized. This is the third aromatic peroxygenase (APO) that has been characterized in detail and the first one that can be produced in high yields. The highest enzyme levels of about 41,000 U l^-1 ^(corresponding to appr. 445 mg l^-1 ^APO protein) exceeded the hitherto reported levels more than 40-fold and were detected in carbon- and nitrogen-rich complex media. The enzyme was purified by FPLC to apparent homogeneity (SDS-PAGE) with a molecular mass of 32 kDa (27 kDa after deglycosylation) and isoelectric points between 4.97 and 5.27. The UV-visible spectrum of the native enzyme showed a characteristic maximum (Soret band) at 418 nm that shifted after reduction with sodium dithionite and flushing with carbon monoxide to 443 nm. The pH optimum of the *M. rotula *enzyme was found to vary between pH 5 and 6 for most reactions studied. The apparent *K_m_-*values for 2,6-dimethoxyphenol, benzyl alcohol, veratryl alcohol, naphthalene and H2O2 were 0.133, 0.118, 0.279, 0.791 and 3.14 mM, respectively. *M. rotula *APO was found to be highly stable in a pH range from 5 to 10 as well as in the presence of organic solvents (50% vol/vol) such as methanol, acetonitrile and *N,N*-dimethylformamide. Unlike other APOs, the peroxygenase of *M. rotula *showed neither brominating nor chlorinating activities.

## Introduction

Enzymes catalyzing oxygen-transfer reactions are of great interest for chemical synthesis since they work selectively and under ambient conditions (Joo et al. 1999). Despite this fact, production of respective biocatalysts is still limited to small scale in the laboratory and the few enzymes available are just provided as expensive fine chemicals. One reason for this is the intracellular nature of oxygenases, which is connected with complex cofactor requirements and low enzyme stability (Urlacher and Eiben 2006). Thus cytochrome P450 monooxygenases (P450s), which represent the most versatile group of oxygen-transferring biocatalysts, need NAD(P)H as electron donating co-substrate and - at least - one accessory protein, a flavin reductase, as electron-transferring partner enzyme (Smith et al. 1994). Most P450s tend to lose their activity rapidly outside the protecting cell, which hampers their purification and use in cell-free reaction systems (Urlacher et al. 2004). Extracellular biocatalysts of the heme peroxidase type could help to overcome this problem, since they are secretory proteins and hence relatively stable, they have the same prosthetic group (protoporphyrine IX = heme) as P450s and form similar activated oxo-intermediates (compound I) (Pickard et al. 1991, McCarthy and White 1983).

In 2004, an extracellular heme-thiolate peroxidase that fulfills these criteria was discovered in the agaric fungus *Agrocybe aegerita *(Ullrich et al. 2004). The enzyme, first described as a haloperoxidase and nowadays mostly referred to as aromatic peroxygenase (APO) or unspecific peroxygenase (EC 1.11.2.1; http://www.chem.qmul.ac.uk/iubmb/enzyme/newenz.html), turned out to be a functional hybrid that shares catalytic properties with peroxidases and monooxygenases (Hofrichter and Ullrich 2006, Hofrichter et al. 2010). With hydrogen peroxide as co-substrate, the enzyme is capable of oxygenating various substrates, among others aromatic rings and alkyl substituents (Ullrich and Hofrichter 2005, Kluge et al. 2007, Aranda et al. 2009), and it cleaves a variety of ethers (Kinne et al. 2009). This direct incorporation of oxygen from H_2_O_2 _can be described as peroxygenation and resembles the so-called peroxide "shunt" known as a side activity of some P450s (Joo et al. 1999).

A second extracellular APO with somewhat different catalytic properties was described for the Ink cap mushroom *Coprinellus radians *(Anh et al. 2007, Aranda et al. 2009, Aranda et al. 2010). Furthermore, various homologous gene sequences and transcripts of putative peroxygenases have been identified in the course of searches in gene databases (Pecyna et al. 2009, Hofrichter et al. 2010).

Using soybean suspensions as growth substrate, moderate amounts (5-10 mg L^-1^) of *A. aegerita *APO (*Aae*APO) and *C. radians *APO (*Cra*APO) can be produced in stirred-tank bioreactors and agitated culture flasks (Ullrich et al. 2004, Anh et al. 2007). High-yield production of wild-type APOs, however, is so far not possible. Therefore, the aim of the present study has been to establish a procedure for the production of high amounts of APO using a novel production organism.

## Material and Methods

### Organism

*Marasmius rotula *[Scop.] Fr. (Pinwheel mushroom) (deposited at the German collection of microorganisms and cell cultures--DSMZ, collection number DSM 25031) was isolated from fruiting bodies that had developed on a meadow near Senftenberg (Germany) containing subsurface woody debris of *Rubinia pseudoacacia *(False Acacia). To confirm the strain affiliation to *M. rotula *at molecular level, the complete internal transcribed spacer (ITS) region including the 5.8S rRNA sequence of the ribosomal DNA was analyzed. For this, the isolated culture was grown on agar containing 1% malt extract and 0.5% glucose. Thereafter, genomic DNA was extracted from 100 mg homogenized fungal mycelium using the DNeasy Plant Mini Kit (Qiagen GmbH, Hilden, Germany) according to the manufacturer's protocol. PCR amplification of genomic DNA was performed in a total volume of 20 μl containing 2 μl template, 9 μl GoTaq Green Master Mix, and 0.4 μl 25 pmol of each of two general fungal ITS-rDNA primers ITS1 and ITS4 (White et al. 1990) using standard cycling conditions. The direct sequencing of resulting 777 bp amplicon cleaned with ExoSAP-IT (USB Europe GmbH, Staufen, Germany) was performed on an ABI PRISM 3730 × l Genetic Analyzer (Applied Biosystems, Darmstadt, Germany) using the Big Dye Terminator v.3.1 Cycle Sequencing Kit (Applied Biosystems). The quality of the obtained sequence was checked by visual inspection of the electropherogram using a Sequence Scanner v.1.0 (Applied Biosystems) and edited using BioEdit v.7.0.9 (Hall 1999). A nucleotide BLAST search at GenBank database (Zhang et al. 2000) in September 2011 revealed several hundred homologous sequences from *Marasmius *and related genera. However, only two sequences in the database reply showed a high query coverage (representing more than 700 aligned nucleotides) and a sequence identity higher than 95%: an unnamed *Marasmius *species (accession number DQ449983, 709 aligned nucleotides, 99% sequence identity) and the only known ITS sequence in GenBank of *M. rotula *(accession number DQ182506, 701 aligned nucleotides, 98% sequence identity) provided by the AFTOL project (*Assembling the fungal tree of life*; Celio et al. 2006). The ITS sequence of *M. rotula *strain DSM 25031 was submitted to GenBank database (accession number JN714927).

### Culture conditions

Fungal stock cultures were grown in culture slants on 2% malt extract agar at 24°C and stored at 4°C in the dark. The content of one slant was homogenized in 40 ml sterile sodium chloride solution (0.9%) and used for the inoculation of liquid cultures (5% vol/vol of the mycelium suspension). The carbon- and nitrogen-rich basic liquid medium contained 28 g L^-1 ^glucose; 12 g L^-1 ^peptone from soybean (Roth, Karlsruhe, Germany) and 3 g L^-1 ^yeast extract (Merck, Darmstadt, Germany) dissolved in distilled water (Ikehata et al. 2004). Enzyme production was performed in 500-mL Erlenmeyer flasks containing 200 mL of the liquid medium. Cultivation occurred on a rotary shaker (120 rpm) at 24°C for three to four weeks. Peroxygenase activity was measured every 1 to 3 days.

*M. rotula *was also cultivated in 5-L stirred-tank bioreactors (Sartorius, Melsungen, Germany) to produce larger amounts of peroxygenase. The medium (4 L) was the same as above and inoculation occurred with 200 ml mycelial suspension (fermentation parameters: 24°C, stirring at 300 rpm and 100% dissolved oxygen without pH-regulation).

### Enzyme assays

Peroxygenase activity was routinely measured by following the oxidation of veratryl alcohol into veratraldehyde (ε_310_: 9,300 M^-1 ^cm^-1^) in McIlvaine buffer at pH 5.5 (Ullrich et al. 2004). Reaction was started by addition of 2 mM H_2_O_2_. Laccase activity in the culture liquid and during the purification was detected by the oxidation of ABTS in McIlvaine buffer at pH 4.5 in the absence of H_2_O_2 _(Majcherczyk et al. 1999).

Further activities of purified *M. rotula *aromatic peroxygenase (*Mro*APO) were measured with benzyl alcohol, 2,6-dimethoxyphenol (DMP) and ABTS under identical conditions by monitoring the formation of benzaldehyde (ε_280_: 1,400 cm^-1 ^M^-1^), dimeric DMP quinone (ε_469_: 49,600 M^-1 ^cm^-1^) and the ABTS cation radical (ε_420_: 36,000 M^-1 ^cm^-1^) (Ullrich et al. 2004). Ring-hydroxylating activity of *Mro*APO was determined by the peroxygenation of naphthalene to 1-naphthol (ε_303_: 2,030 M^-1 ^cm^-1^). The assay mixture contained in a total of 2 ml: 1 ml sodium phosphate/citrate buffer (100 mM pH 5.5), 200 μl naphthalene (5 mM) dissolved in 100% acetonitrile and 10 to 100 μl enzyme solution. The reaction was started with 20 μl H_2_O_2 _(200 μM) (Kluge et al. 2007).

### Enzyme purification

The culture liquid of *M. rotula *was separated from the mycelium by filtration through paper filters (GF8, Whatman GmbH, Dassel, Germany). The filtrate was frozen at -80°C and then re-thawed to precipitate and remove extracellular glucans. After thawing, the culture liquid was filtrated through glass fiber filters (GF 8, Whatman).

The filtrate was concentrated 40-fold by two steps of ultrafiltration using two tangential-flow cassettes (Sartocon Slice Cassette, Hydrosart, cut-off 10 kDa, Sartorius, and Omega membrane, cut-off 10 kDa, Pall Life Sciences, Dreieich, Germany). All subsequent chromatographic purification steps were performed with an Äkta FPLC™System (GE Healthcare Europe GmbH, Freiburg, Germany). In the first step, the crude preparation was loaded onto a Q Sepharose FF column (anion exchanger XK 26/20, GE Healthcare) and the proteins were eluted with a linear gradient of 0-0.7 M NaCl in 10 mM sodium acetate buffer (pH 5.5) at a flow rate of 6 ml min^-1^. *Mro*APO containing fractions were pooled, concentrated and dialyzed against 10 mM sodium acetate (pH 5.5, 10 kDa cut-off Omega membrane, Pall Life Sciences). Pooled fractions were subjected to a second anion exchanger consisting of mono beads (Mono Q 10/100, GE Healthcare) and using sodium acetate (10 mM, pH 5.5) and an increasing gradient up to 0.4 M sodium chloride for elution. *Mro*APO containing fractions were pooled, concentrated and dialyzed against 10 mM sodium acetate. In the next step, an anion exchanger consisting of Source 15Q (10/10, GE Healthcare) was used along with sodium acetate (25 mM, pH 5.0) and an increasing gradient up to 0.4 M sodium sulphate for elution. *Mro*APO containing fractions were pooled, concentrated and dialyzed against 50 mM sodium phosphate buffer (pH 8.0, 10 kDa cut-off Vivaspin 20, Satorius Stedim Biotech GmbH, Goettingen, Germany). Afterwards, size exclusion chromatography (SEC; Superdex 75 16/60, GE Healthcare) was carried out under isocratic conditions in 50 mM sodium phosphate (pH 8.0, +0.15 M sodium chloride). The pooled and concentrated *Mro*APO fractions were re-chromatographed on a Mono Q column (5/50, GE Healthcare) using sodium acetate (10 mM, pH 5.5) and an increasing gradient up to 0.25 M sodium chloride for elution. *Mro*APO containing fractions were eventually pooled, concentrated and dialyzed against 50 mM sodium phosphate buffer (pH 8.0, 10 kDa cut-off Vivaspin 20) and stored at 4°C. During early FPLC-separation steps (e.g. first Mono-Q separation), the elution profiles showed up to four heme peaks with veratryl alcohol oxidizing activity; we focused our purification efforts always on the most active fraction (i.e. that with the highest specific activity).

### Characterization of *Mro*APO

Molecular mass of purified *Mro*APO was determined by sodium dodecyl sulfate-polyacrylamide gel electrophoresis (SDS-PAGE) using a 12.5% NuPage Novex Bis-Tris Gel (Invitrogen, Darmstadt, Germany). A low-molecular mass protein calibration kit (MBI Fermentas, PageRuler™, Roth, Germany) was used as standard. Isoelectric focusing (IEF) was carried out using precast gels (pH 3-7; Invitrogen) and specific IEF markers (pH 3-10; Serva, Heidelberg, Germany) as standard. Electrophoretically separated protein bands were visualized with the Colloidal Blue staining kit (Invitrogen).

Purified *Mro*APO (75 μg) was denaturated with SDS and deglycosylated for 4 hours with the Enzymatic Protein Deglycosylation Kit from Sigma (Saint Louis, MO, USA). The latter contained PNGase F, *O*-glycosidase, two α-2(3,6,8,9) neuramidases as well as β-1,4-galactosidase and β-*N*-acetylglucosaminidase and was used according to the instructions of the provider. Molecular mass of the deglycosylated protein was determined by SDS-PAGE using a 12% NuPAGE Novex Bis-Tris Gel (Invitrogen).

Apparent Michaelis-Menten (K_m_) and catalytic (k_cat_) constants of purified *Mro*APO were determined at pH 5.5 for veratryl alcohol, benzyl alcohol, DMP, ABTS, naphthalene, and H2O2. Lineweaver-Burk plots were prepared from the initial rates obtained with various substrate concentrations while the concentration of the second substrate was kept constant.

Stability of *Mro*APO in organic solvents was tested by incubating the enzyme (1 U = 0.31 μM) in aqueous buffer mixtures (vol/vol) of methanol, acetonitrile and *N,N*-dimethylforamide (DMF). Organic solvent concetrations of 10, 30, 50 and 70% were used. All mixtures were kept at 24°C for up to 120 min. For determination of the remaining enzyme activity, samples were taken after 1, 30, 60 and 120 min and measured using the veratryl alcohol assay mentioned above.

The UV-Vis spectrum of the resting-state *Mro*APO was recorded in 10 mM sodium acetate buffer (pH 5.5) in the range from 200 to 700 nm using a Cary 50 spectrophotometer (Varian, Darmstadt, Germany). To obtain the reduced CO-enzyme complex, samples were reduced with sodium dithionite and flushed with carbon monoxide for 2 min.

For N-terminal analysis, *Mro*APO was separated by SDS-PAGE as described above and transferred from the SDS gel to a polyvinylidene fluoride membrane (Amersham Biosciences, Freiburg, Germany) by electroblotting. Sequencing by Edman degradation and *de-novo*-peptide sequencing using matrix-assisted laser desorption/ionization mass spectrometry (MALDI-TOF/TOF) after digestion with trypsin were performed by Protagen AG (Dortmund, Germany). Sequences were compared with known APOs (*A. aegerita, C. radians*) and chloroperoxidase (CPO; *Caldariomyces fumago*) as well as with putative APO- and CPO-like sequences from the National Center for Biotechnology Information (NCBI) GenBank database by using BLASTp.

### Further substrate oxidation studies

Enzymatic conversion of toluene was performed in 2-mL reaction vials containing citrate phosphate buffer (pH 5.5), 1 mM toluene, and 10 U *Mro*APO (3.1 μM). The reaction was started by addition of H2O2 (2 mM), which was repeated four times in 2.5 min steps.

For naphthalene oxidation, two reaction solutions were prepared. Solution A contained 2 U *Mro*APO (0.62 μM) in 1 mL citrate phosphate buffer (pH 5.5) and solution B consisted of 2 mM naphthalene and 2 mM H2O2 in 1 mL 50% (vol/vol) acetonitrile. Every 2.5 min, 250 μL of solution B were transferred to solution A. After 10 min, reactions were stopped by addition of 20 μl HCl (37%).

Reaction products were analyzed by HPLC using an Agilent^® ^1200 system (Waldbronn, Germany) equipped with a diode array detector and a LiChrospher reversed phase (C18) column (4.6 × 125 mm, 5 μm; Phenomenex, Aschaffenburg, Germany). For toluene and its oxidation products, a gradient separation was applied from 15 to 80% acetonitrile (0-5 min; 15%; 5-25 min 15-80%) in 20 mM aqueous potassium dihydrogenphosphate buffer (pH 2.8) at a flow rate of 0.7 mL min^-1^. For naphthalene, a gradient from 20 to 80% acetonitrile (0-5 min; 20%; 5-20 min; 20-80%) in 20 mM potassium dihydrogen phosphate (pH 2.8) was used at a flow rate of 1 mL min^-1^. Eluted substances were recorded at 220 nm and identified/quantified by means of authentic standards.

Possible halogenating activity of *Mro*APO was tested as previously described (Ullrich and Hofrichter 2005). Briefly, the enzyme (0.31 μM) was incubated in potassium phosphate buffer (100 mM, pH 3) in the presence of phenol (0.1 mM), potassium bromide or chloride (10 mM) and H_2_O_2_. After 10 min, the reaction mixture was analyzed by HPLC for the formation of bromo- and chlorophenols using authentic monohalophenols as standards. In a second test, the oxidation of bromide (Br^-^) and iodide (I^-^) into tribromide (Br_3_^-^) and respectively triodide (I_3_^-^) was spectrophotometrically followed (Libby et al. 1982).

### Chemicals

2,2'-Azinobis-(3-ethylbenzothiazoline-6-sulfonate) (ABTS) was purchased from Applichem (Darmstadt, Germany), 2,6-dimethoxyphenol (DMP), naphthalene, 1-napththol, 2-naphthol, potassium chloride and toluene from Sigma-Aldich (München, Germany). Veratryl alcohol, veratraldehyde, veratric acid, benzyl alcohol, benzaldehyde, benzoic acid, phenol, H2O2 (30%, w/v), sodium acetate, sodium chloride, sodium sulphate, methanol and acetonitrile were obtained from Roth (Steinheim, Germany). All other chemicals and solvents were purchased from Merck (Darmstadt, Germany).

## Results

### Enzyme production

**Figure 1:**
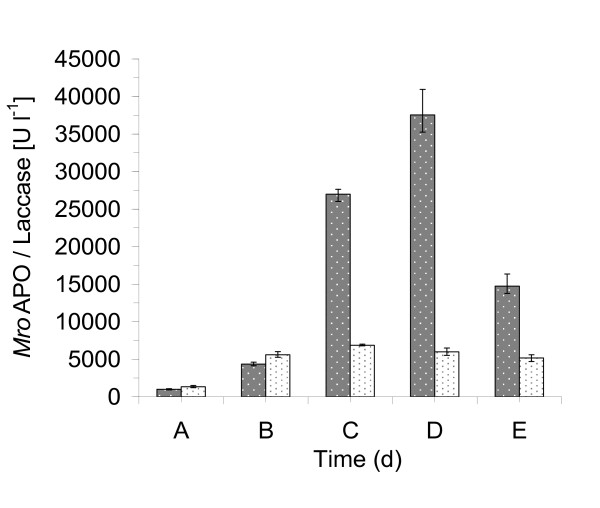
**Effect of different combinations of glucose, soybean peptone and yeast extract on the production of *Mro*APO (*grey columns with white dots*) and laccase (*white columns with black dots*)**. A - glucose (14 g L^-1^), soybean peptone (6 g L^-1^), yeast extract (1.5 g L^-1^), B - glucose (28 g L^-1^), soybean peptone (12 g L^-1^), yeast extract (3 g L^-1^), C - glucose (28 g L^-1^), soybean peptone (48 g L^-1^), yeast extract (3 g L^-1^), D - glucose (42 g L^-1^), soybean peptone (48 g L^-1^), yeast extract (4.5 g L^-1^), E - glucose (56 g L^-1^), soybean peptone (48 g L^-1^), yeast extract (6 g L^-1^). Cultivation was carried out in 500-ml flasks containing 200 ml of the respective liquid medium. Flasks were shaken at 120 rpm at 24°C in the dark for 28 days (data points represent maximum levels obtained within the cultivation period). *Mro*APO activity was measured with veratryl alcohol at pH 5.5 (modified according to Ullrich et al. 2004). Laccase activity was measured with ABTS at pH 4.5 (Majcherczyk et al. 1999). Dry masses after 28 days: A - 6.35 g L^-1^, B - 12.15 g L^-1^, C - 17.5 g L^-1^, D - 25.8 g L^-1^. E - 15.5 g L^-1^. Values given are means of three parallel flasks with standard deviations.

**Figure 2:**
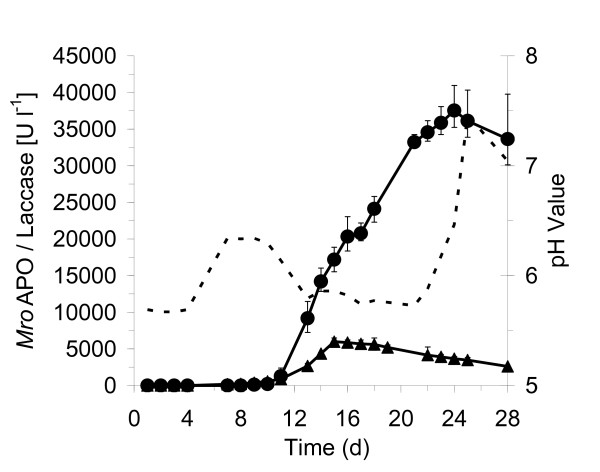
**Time course of peroxygenase (*circles*) and laccase (*triangles*) production by agitated cultures of *M. rotula *under optimized conditions in a liquid medium consisting of 42 g L^-1 ^glucose, 45 g L^-1 ^soybean peptone and 4.5 g L^-1 ^yeast extract**. *Mro*APO activity was measured with veratryl alcohol at pH 5.5 and laccase activity with ABTS at pH 4.5. Data points represent mean values of three parallel culture flasks with standard deviations. The dotted line marks the time course of pH.

A medium containing both glucose, peptone from soybean, and yeast extract was essential for the production of *Mro*APO. Increasing the amount of these components resulted in a considerable enhancement of enzyme activity. Their concentrations were varied from 100% to 400% and respectively 800%, i.e. 14 to 56 g L^-1 ^glucose, 6 to 48 g L^-1 ^soybean peptone and 1.5 to 6 g L^-1 ^yeast extract were tested (Figure 1). The maximum activity of *Mro*APO (40,944 U L^-1^, veratryl alcohol assay) was detected in the presence of 42 g L^-1 ^glucose (300%), 48 g L^-1 ^soybean peptone (700%) and 4.5 g L^-1 ^yeast extract (300%) and later (after purification) found to correlate with roughly 445 mg L^-1 ^peroxygenase protein. Figure 2 describes the time course of *Mro*APO activity and pH under these conditions. During the first days of cultivation, the pH increased slightly from 5.6 to 6.3 on day 8. *Mro*APO activity appeared in the culture liquid 10 to 11 days after inoculation and within this phase, the pH decreased from pH 6.3 to pH 5.7 on day 12 and remained almost constant until day 21 (Figure 2). The maximum activity of *Mro*APO was detected on day 24 of cultivation, i.e. 3 to 4 days after the pH had started to increase again reaching a maximum value of 7.5 on day 25. Afterwards, both the enzyme activity and the pH slightly dropped (~10%) until the end of the experiment (day 28) (Figure 2). The fungal biomass consisting of whitish pellets (max. 3-5 mm in diameter) at this time was 25.8 g L^-1 ^(dry mass). In addition to peroxygenase, also laccase activities were detected with a maximum of 5,992 U L^-1 ^on day 15 (Figure 2).

To obtain larger amounts of *Mro*APO, *M. rotula *was cultured in 5-L stirred-tank bioreactors using the optimized medium mentioned above. Production of *Mro*APO started on day 12 of fermentation and reached the maximum of 25,000 U L^-1 ^(280 mg L^-1^) on day 24 (data not shown); immediately afterwards, the culture liquid of the bioreactors was harvested and used for enzyme purification studies. The final biomass in the bioreactor was 45.4 g L^-1^.

### Purification and physical characterization of *Mro*APO

**Figure 3:**
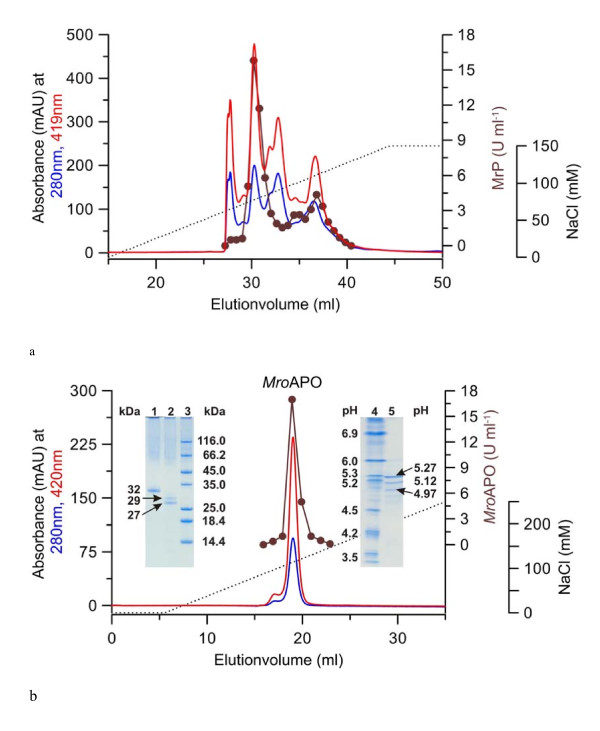
**Purification of *Mro*APO by FPLC (Mono Q, steps two and five)**. (A) - Early FPLC separation step on a Mono Q column 10/100 column showing four major heme peaks with veratryl alcohol oxidizing activity (further purification focused on the second peak). (B) - FPLC elution profile of the final purification step of *Mro*APO performed on a Mono Q 5/50 column. Heme absorption at 420 nm (*red line*), total protein at 280 nm (blue line), NaCl gradient (*dotted line*). APO activity was detected by the oxidation of veratryl alcohol at pH 5.5. The insets show the respective SDS-PAGE (left) and isoelectric focusing gels (right). The second lane of the SDS-PAGE gel shows the molecular mass of the partially and fully deglycosylated *Mro*APO protein at 29 and 27 kDa, respectively.

The culture liquid was separated from the fungal mycelium by filtration, then frozen at -80°C, re-thawed and centrifuged to remove polysaccharides (Ullrich et al. 2004). A loss of enzyme activity was not caused by this procedure. Afterwards, the crude extract was concentrated by ultrafiltration and separated by five chromatographic steps using an FPLC system and strong anion exchangers (Q Sepharose FF, Mono Q 10/100, Source 15Q, Mono Q 5/50 columns) as well as a molecular sieve (SEC Superdex column) (Table 1). Figures 3A and 3B show the elution profiles of an early Mono-Q 10/100 separation with four pronounced peroxygenase peaks and the final purification step on a Mono Q 5/50 column with just one peroxygenase fraction. The specific activity of the latter was 77 U mg^-1 ^(veratryl alcohol assay) and it was used for the physical and catalytic characterization of the protein.

**Table 1:** Exemplary table for the purification of *Mro*APO by fast protein liquid chromatography (FPLC)

Steps	Total Activity (Units)	Total Protein (mg)	Specific Activity (U mg^-1^)	Yield (%)	Purification (fold)
Culture fluid	11,700	730	16	100	1
Ultrafiltration step	11,520	415	28	98	1.7
Q Sepharose FF	10,413	218	48	89	3.0
Mono Q	6,235	118	53	53	3.3
Source 15Q	1,457	22	88	17	5.5
SEC	768	9	90	7	5.6
Mono Q	75	0.98	76	0.7	4.8

Enzyme activities are based on the oxidation of veratryl alcohol to veratrylaldehyde at pH 5.5. The purification processes started with 468 mL culture fluid from the bioreactor fermentation (volume activity 25 U mL^-1^) corresponding to a total of 11,700 Units.

SDS-PAGE revealed a molecular mass of 32 kDa for the purified *Mro*APO protein and analytical isoelectric focusing gave three pI-bands between 4.97 and 5.27 (major band at 5.27; inset of Figure 3). The purified and re-chromatographed major isoform was deglycosylated to remove covalently bound carbohydrates. The deglycosylated protein had a molecular mass of 27 kDa, i.e. the carbohydrate content of the mature protein is 16% (Figure 3).

**Figure 4:**
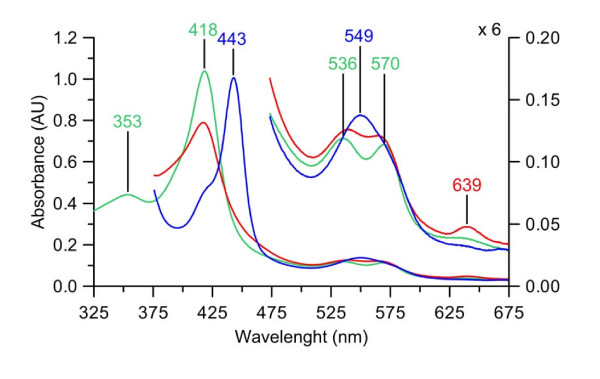
**UV-Vis absorption spectra of purified *Mro*APO**. Native *Mro*APO (green), *Mro*APO reduced with dithionite (red) and reduced CO-complex of *Mro*APO (blue).

The UV-visible spectrum of the resting state enzyme showed a characteristic absorbance at 418 nm (Soret band), two further maxima of at 570 nm 536 nm (α- and β-bands) as well as a δ-band at 353 nm. After addition of sodium dithionite and flushing with CO, the CO-complex of reduced *Mro*APO was formed. This was accompanied by a shift of the Soret band to 443 nm, which is still in the characteristic range of heme-thiolate proteins (Figure 4). The spectral data of *Mro*APO in comparison to four other heme-thiolate proteins are given in Table 2.

**Table 2:** Spectral characteristics of *Mro*APO in comparison to respective data of two other APOs, CPO and a P450 monooxygenase (Hofrichter and Ullrich 2006, Anh et al. 2007, Faber et al. 2001)

Organism	Enzyme	Soret maxima (nm)			Further maxima of the resting enzyme		
		
		Resting	Reduced	CO adduct	α	β	δ
*Marasmius rotula*	*Mro*APO	418	418	443	570	536	353
*Agrocybe aegerita*	*Aae*APO	420	413	447	572	540	359
*Coprinus radians*	*Cra*APO	422	426	446	571	542	359
*Caldariomyces fumago*	CPO	401	409	443	-	545	-
*Aspergillus niger*	CYP53A1	418	n.d.	448	568	538	361

**Figure 5:**
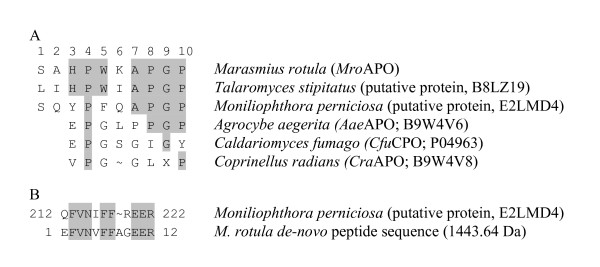
***N*-terminal sequences (A) and peptide fragment alignment (B) of *Mro*APO, *Aae*APO, *Cra*APO and *Cfu*CPO as well as hypothetical APO-like sequences**. UniProt protein sequence accessions are given in brackets.

The obtained N-terminal sequence of *Mro*APO (10 amino acids) showed 40%, 20% and 20% identity to the N-terminal sequences of *Aae*APO, *Cra*APO and *Cfu*CPO (Figure 5A). An additional NCBI database search with MS/MS data for tryptic peptides of purified *Mro*APO failed, and therefore *de-novo *peptide sequencing was performed (Figure 5B). The data obtained for an *Mro*APO peptide consisting of 12 amino acids show 67% identity to the putative APO-like protein sequence of *Moniliophthora perniciosa *(gi 215458597) that is also a member of the family Marasmiaceae and closely related to the genus *Marasmius *(Kerekes and Desjardin 2009). However, the second *de-novo *peptide obtained (STTPITIPLLTTGIHR, 1720.97 Da) did not show any similarity to known fungal peroxidases/peroxygenases according to respective database searches.

### Oxidation of different substrates, influence of pH and kinetic parameters

**Figure 6:**
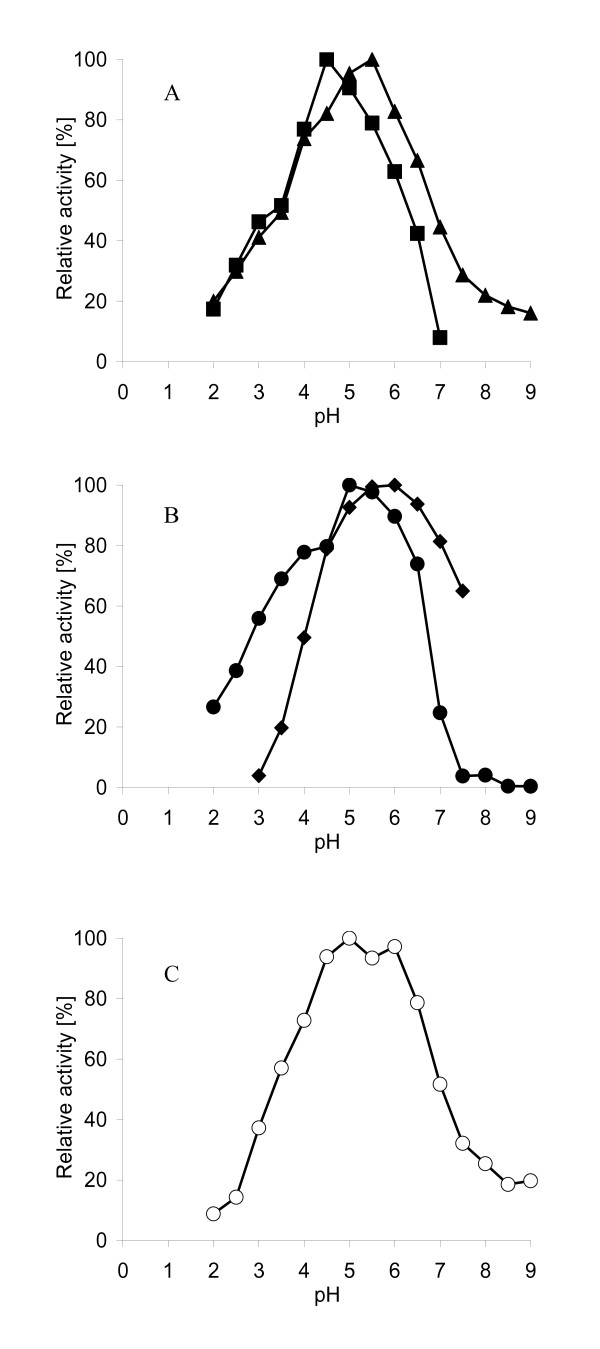
**Effect of the pH on the *Mro*APO-catalyzed oxidation of different substrates**. A - ABTS (0.6 mM) (*squares*) and veratryl alcohol (5 mM) (*triangles*), B - DMP (6 mM) (*diamonds*) and naphthalene (2 mM) (*black circles*), C - benzyl alcohol (*white circles*). ABTS was oxidized into the corresponding cation radical (ABTS^+·^), vertatryl alcohol into vertraldehyde, DMP into a dimeric DMP-quinone, naphthalene into 1-naphthol and benzyl alcohol into benzaldehyde. Reactions were performed in sodium phosphate/citrate buffer in the presence of 2 mM H_2_O_2 _at 25°C. The data are means of three parallel experiments with standard deviation < 5%.

The pH dependence of *Mro*APO was studied using DMP, benzyl alcohol, veratryl alcohol, ABTS and naphthalene as substrates (Figure 6). All pH profiles have pronounced acidic maxima between 4.5 and 6. Thus, the optimum of ABTS oxidation was found to be pH 4.5, those of veratryl alcohol and DMP oxidation pH 5.5 and 5.0, respectively. The oxidation of benzyl alcohol showed two distinct maxima at pH 5 and 6. The optimum for the hydroxylation of naphthalene into 1-naphthol (major product) was detected between pH 5.5 and 6.0. All in all, *Mro*APO was active in a broad pH range between 2 and 9.

Apparent Michealis-Menten (K_m_) and catalytic constants (k_cat_) as well as catalytic efficiencies (k_cat_/K_m_) of all tested substrates are summarized in table 3. Under the conditions used, DMP and benzyl alcohol were the best substrates; veratryl alcohol and naphthalene were oxidized with 3- and 18-times lower catalytic efficiencies, respectively. While the turnover numbers for all substrates were roughly in the same order of magnitude (25 to 187 s^-1^), the K_m _values varied in one order of magnitude from 0.071 mM (for ABTS) to 0.791 mM (for naphthalene). Interestingly, the highest K_m _(3.14 mM) was determined for H_2_O_2 _(with veratryl alcohol as substrate) indicating a relatively low affinity of the enzyme to its electron-accepting co-substrate.

**Table 3:** Apparent kinetic parameters of purified *Mro*APO

Substrate	pH	**K**_**m**_ (mM)	**k**_**cat**_ **(s**^**-1**^**)**	**k**_**cat**_**/K**_**m**_ (s^-1 ^M^-1^)
ABTS	4.5	0.071	25	3,53*10^5^
DMP	5.5	0.133	70	5.29*10^5^
Benzyl alcohol	5.5	0.118	62	5.29*10^5^
Veratryl alcohol	5.5	0.279	49	1.76*10^5^
Naphthalene	5.5	0.791	33	4.25*10^4^
H_2_O_2_	5.5	3.14	76	2.42*10^4^

Reactions were performed in sodium phosphate/citrate buffer (pH 4.5 or 5.5) in the presence of 2 mM H_2_O_2 _at 25°C. The K_m _for the co-substrate H_2_O_2 _was determined in the presence of 5 mM veratryl alcohol. The data are means of three parallel experiments with standard deviation < 5%.

Using 0.05-0.4 U (0.0155-0.124 μM) of *Mro*APO, the optimum concentration of H_2_O_2 _for the oxidation of veratryl alcohol was found to be 4 mM; at a concentration of 2 mM peroxide, the detected activity was by 15% lower. In case of benzyl alcohol and DMP oxidation, the optimum concentration of H2O2 was over 10 mM and 5 mM, respectively, indicating a rather high co-substrate requirement in these reactions and possibly a pseudo-catalase activity that destroys/consumes H_2_O_2 _without contributing to the peroxygenase cycle. In contrast, the hydroxylation of naphthalene was optimal when a lower amount of 1 mM H2O2 was used (data not shown).

The influence of organic solvents on the enzyme stability was determined in aqueous buffer mixtures at concentrations of 10, 30, 50 and 70% vol/vol using the veratryl alcohol assay. In case of methanol, at least 80% of the initial activity was still found after 2 hours of incubation at all concentrations. A 2-hour incubation in the presence of 10 or 30% acetonitrile did not cause any decrease in *Mro*APO activity, while 50 and 70% acetonitrile caused an activity loss of 23 and 28%, respectively. Concentrations of 10, 30 or 50% DMF did not affect the activity of *Mro*APO but it decreased by more than 90% within the first 30 min of incubation at 70% DMF.

### Further substrate oxidation studies

To further characterize the oxygen transfer catalyzed by *Mro*APO, the product patterns and ratios of toluene and naphthalene oxidation were analyzed. Using toluene (1 mM), the major oxidation product was benzoic acid (0.896 mM), followed by benzaldehyde (0.074 mM) and methyl-*p*-benzoquinone (0.037 mM); the former two products were the result of side chain oxidation while the formation of the latter can only be explained by an initial hydroxylation in *para*-position to give *p*-cresol that in turn was oxidized into the corresponding *p*-benzoquinone derivative. On this basis, a ratio of side chain vs. ring hydroxylation of 26:1 can be inferred for toluene oxidation by *Mro*APO. Naphthalene (1 mM) was regioselectively converted into 1-naphthol (0.922 mM) and 2-naphthol (0.078 mM) as major and minor products, respectively (ratio 12:1).

*Mro*APO was neither capable of brominating nor chlorinating phenol in the presence of potassium bromide or respectively potassium chloride, i.e. no bromo- or chlorophenol was detectable by HPLC. The assay was performed both at pH 3 and pH 5.5 and in the presence of different peroxide concentrations. Also the formation of tribromide, as the result of possible bromide oxidation, was not observed. Merely iodide was slowly oxidized into triiodide. These results indicate a weak halogenating activity of *Mro*APO that sets the enzyme apart from other fungal heme-thiolate peroxidases (CPO, *Aae*APO) (Hofrichter and Ullrich 2006).

## Discussion

The agaric basidiomycete *M. rotula *produced a novel aromatic peroxygenase (*Mro*APO) during growth in agitated liquid culture. The enzyme was purified to apparent homogeneity and characterized. It is a relatively small (M_w_: 32 kDa), glycosylated (16%) heme-thiolate protein that peroxygenates naphthalene, toluene, benzyl and veratryl alcohol as well as oxidizes typical peroxidase substrates such as ABTS and DMP (but neither chloride nor bromide). The fungus produced up to 41.000 U L^-1 ^(445 mg L^-1^) of *Mro*APO in a complex medium rich in organic carbon and nitrogen (4.2% glucose, 4.5% soybean peptone and 0.45% yeast extract). To our best knowledge, the calculated amount of 445 mg L^-1 ^peroxygenase protein is one of the highest levels of a secreted heme peroxidase reported for a wild-type basidiomycete so far.

Interestingly, culturing the known peroxygenase producers *A. aegerita *and *C. radians *in the same complex medium did neither promote growth nor enzyme secretion (Gröbe 2011, unpublished result). *A. aegerit*a was the first APO producer described and found to secrete appreciable levels of the enzyme, *Aae*APO^a^, merely in the presence of soybean meal (Ullrich et al. 2004). The best result, 2,021 U L^-1 ^corresponding to 12 mg L^-1 ^APO protein, was reported for an agitated slurry-culture containing 60 g L^-1 ^soybean meal. However, due to the high particle density, usually lower amounts of soybean meal (20 g L^-1^) have been used to produce *Aae*APO in stirred-tank bioreactors with activity levels of 500 to 1,500 U L^-1 ^(2.5-10 mg L^-1^) (Ullrich et al. 2004, Ullrich et al. 2009). The second APO producer, *C. radians*, secreted 176 U L^-1 ^(4.5 mg L^-1^) in a similar soybean medium (Anh et al. 2007). As in case of *Mro*APO, supplementation of this medium with glucose was found to further stimulate *Cra*APO production; the best result (277 U L^-1^, ~7 mg L^-1^) was obtained when 3% soybean meal and 4% glucose were used (Anh et al. 2007). Using *C. fumago *in a semi-continuous flow bioreactor, levels of *Cfu*CPO greater than 600 mg L^-1 ^(maximum level in one occasion 1,083 mg L^-1^) were obtained in a synthetic medium based on glucose as carbon source (Blanke et al. 1989). Batch fermentation of the same fungus in an optimized fructose-based medium resulted in *Cfu*CPO levels of up to 450 mg L^-1 ^(Carmichael and Pickard 1989). The maximum *Mro*APO levels reported here are in the same order of magnitude (up to 445 mg L^-1 ^in culture flasks, and 280 mg L^-1 ^in stirred-tank bioreactors), which is remarkable, since *M. rotula *is an agaric basidiomycete (known to be sensitive and slowly growing; Ullrich et al. 2004, Tang et al. 2007), whereas *C. fumago *is a fast growing ascomycete (sooty mold). To summarize, protein yields of heme-thiolate peroxidases follow this order:

CfuCPO≈MroAPO>>AaeAPO >CraAPO

Production of a classic peroxidase (EC 1.11.1.7) by *Coprinopsis (Coprinus) cinerea *UAMH 4103 in a similar complex medium (2.9% glucose, 1.4% peptone, 0.3% yeast extract, 0.3% malt extract) resulted in activities up to 68,000 U L^-1 ^(determined with a colorimetric assay using phenol and 4-aminoantipyrine, and corresponding to 155 mg L^-1 ^protein; Ikehata et al. 2004, Ikehata et al. 2005).

With a molecular mass of 32 kDa, *Mro*APO is considerably (by 10-40%) smaller than other fungal heme-peroxidases including APOs (43-46 kDa) and *Cfu*CPO (40-46 kDa) (Hatakka 1994, Hofrichter et al. 2010). Furthermore *Mro*APO is less glycosylated (16%) than the other heme-thiolate peroxidases (*Cra*APO 37%, *Aae*APO 20%, CPO 25-30%). The determined isoelectric points of *Mro*APO varied between 4.97 and 5.27 (major band at 5.27) indicating the presence of several similar isoforms. Isoforms with the same molecular mass but slightly different, acidic pIs were also reported for *Aae*APO (4.6-5.6, Ullrich et al. 2004; 5.2-6.1, Ullrich et al. 2009) and *Cra*APO (3.8-4.2, Anh et al. 2007).

The UV-Vis absorption spectrum of *Mro*APO showed a characteristic shift of the Soret band from 418 nm to 443 nm after reduction and CO-treatment. The shift of the CO-complex' Soret band towards 450 nm is characteristic for all heme-thiolate enzymes (Omura 2005), including APOs, *Cfu*CPO and P450s.

A distinctive catalytic feature of *Mro*APO is the limited halide oxidation (restricted to iodide) and hence the lacking of brominating or chlorinating activities. In contrast, *Aae*APO and *Cra*APO can efficiently oxidize bromide and *Cfu*CPO in addition chloride (Hofrichter and Ullrich 2006), i.e. halide oxidation by heme-thiolate peroxidases follows this order:

CfuCPO >> AaeAPO≈CraAPO>MroAPO

On the other hand, the catalytic activities of *Mro*APO towards classic peroxidase substrates such as ABTS and DMP as well as the peroxygenation of aryl alcohols resemble reactions catalyzed by the three other heme-thiolate peroxidases (*Aae*APO, *Cra*APO and *Cfu*CPO; Ullrich et al. 2004, Anh et al. 2007, Baciocchi et al. 2001). Unlike *Cfu*CPO - but in accordance with *Aae*APO and *Cra*APO - *Mro*APO was found to hydroxylate naphthalene and the aromatic ring of toluene (Zaks and Dodds 1995, Ullrich and Hofrichter 2005, Anh et al. 2007), and thus its classification as APO is justifiable (Hofrichter et al. 2010). Naphthalene hydroxylation by *Mro*APO is about 10-times less efficient than by *Aae*APO but comparable to *Cra*APO (Kluge et al. 2007, Anh et al. 2007). In case of toluene conversion by *Mro*APO, the observed ratio of ring vs. methyl group oxidation of 26:1 is considerably higher than that of *Aae*APO (2:1; Ullrich et al. 2009) and points to a preference of alkyl side-chains (e.g. benzylic carbon) over aromatic rings in peroxygenation reactions. A similar preference of non-aromatic carbon was observed for methylnaphthalenes and fluorene oxidation by *Cra*APO while *Aae*APO again primarily hydroxylated the aromatic rings (Aranda et al. 2010). In summary, the oxygen transfer potential of heme-thiolate peroxidases can be arranged in the following order:

AaeAPO>CraAPO≈MroAPO>CfuCPO

The optimum H_2_O_2 _concentration for the oxidation of veratryl alcohol by *Mro*APO was 4 mM (for benzyl alcohol even > 10 mM), which clearly exceeds the values reported for *Cra*APO (0.5-0.7 mM) and *Aae*APO (2 mM) (Anh et al. 2007, Ullrich et al. 2004). This fact is also reflected by a 2-3 times lower affinity of *Mro*APO to H_2_O_2 _(K_m_: 3.1 mM) and implies a generally higher peroxide demand that may be caused by a pseudo-catalase activity. On the other hand, this K_m _is in the same order of magnitude as the values for *Aae*APO and *Cra*APO (1.3 and 1.2 mM, respectively; Ullrich et al. 2004, Anh et al. 2007). *Mro*APO was found to be active in a wide pH range between 2 and 9. This behavior generally resembles *Aae*APO and *Cra*APO, but unlike the latter, *Mro*APO did not show a neutral but an acidic pH optimum (between 5.5 and 6) in case of naphthalene hydroxylation (Kluge et al. 2007, Anh et al. 2007).

*Mro*APO is the third aromatic/unspecific peroxygenase that has been characterized. Concerning its most relevant catalytic property - the transfer of peroxide-borne oxygen to non-activated carbon - it ranges, together with *Cra*APO, between *Aae*APO and *Cfu*CPO. The high stability towards pH and organic solvents as well as the possibility to "naturally over-express" *Mro*APO, i.e. to obtain enzyme yields of several hundred mg L^-1 ^using a wild-type strain, makes the enzyme and the fungus promising targets of further bioengineering and molecular studies, for example, regarding peroxygenase production at larger scale, promotor analysis and heterologous expression of low-yield peroxygenases from other basidiomycetes.

## Competing interests

The authors declare that they have no competing interests.

## Endnotes

^a ^In earlier publications, the enzyme was abbreviated AaP (*Agrocybe aegerita *peroxidase/peroxygenase; Ullrich et al. 2004, Hofrichter and Ullrich 2006)
